# Insulin‐Like Growth Factor Binding Protein 2 Drives Neurodegeneration in Parkinson's Disease: Insights From In Vivo and In Vitro Studies

**DOI:** 10.1111/cns.70076

**Published:** 2024-10-16

**Authors:** Jing An, Lulu Wen, Haiyang Yu, Zhongqi Bu, Juan Feng

**Affiliations:** ^1^ Department of Neurology Shengjing Hospital of China Medical University Shenyang Liaoning China

**Keywords:** apoptosis, insulin‐like growth factor binding protein 2, mitochondria dysfunction, oxidative stress, Parkinson's disease

## Abstract

**Aims:**

Insulin‐like growth factor binding protein 2 (IGFBP2) is implicated in various neurodegenerative diseases. However, its role in Parkinson's disease (PD) is unclear.

**Methods:**

PD rat model was established by 6‐OHDA injection. After 3 weeks, mRNA‐seq was conducted. Rats received rIGFBP2 via intra‐MFB injection 6 h prior to 6‐OHDA infusion, and the effect of IGFBP2 in PD rats was investigated by western blotting, IHC, specific kits, JC‐1 staining, and TUNEL analysis. In vitro, PC12 cells were treated with 6‐OHDA, and CCK‐8, specific kits, Hoechst 33258 staining, Western blotting, and JC‐1 staining were performed to assess the IGFBP2's role.

**Results:**

mRNA‐seq revealed DEGs in PD, with attention to downregulated IGFBP2. rIGFBP2 treatment aggravated neurobehavioral deficits, decreased TH expression, Ψm, ATP level and SOD, GSH‐Px activities but increased α‐synuclein, ROS, MDA, mitochondrial cytochrome *c* contents, cell apoptosis in 6‐OHDA‐lesioned rats, which might be mediated through inactivating IGF‐1R/AKT pathway. In 6‐OHDA‐treated PC12 cells, rIGFBP2 aggravated cell injury, demonstrated by decreased cell viability and increased apoptosis, oxidative stress, and mitochondrial dysfunction. Co‐treatment with rIGFBP2 and rIGF‐1 partially reversed the effect of rIGFBP2 on cell damage.

**Conclusion:**

IGFBP2 exacerbates neurodegeneration in PD through increasing oxidative stress, mitochondrial dysfunction, and apoptosis via inhibiting IGF‐1R/AKT pathway.

## Graphical abstract

The study demonstrates that rIGFBP2 treatment exacerbates Parkinson's disease through increasing oxidative stress, mitochondrial dysfunction, and apoptosis via the IGF‐1/IGF‐1R signaling pathway.

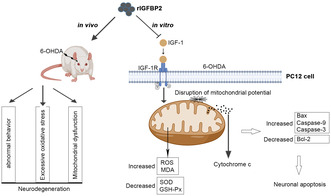

**Abbreviations**
6‐OHDA: 6‐hyhydroxydopamine
ATP: adenosine triphosphate
CCK‐8: cell counting kit‐8
DEGs: differentially expressed genes
GSH‐Px: glutathione peroxidase
IGF‐1: insulin‐like growth factor‐1
IGFBP2: insulin‐like growth factor binding protein 2
IHC: immunohistochemistry
MDA: malondialdehyde
mRNA‐seq: mRNA sequencing
PD: Parkinson's disease
rIGF‐1: recombinant IGF‐1
rIGFBP2: recombinant IGFBP2
ROS: reactive oxygen species
RT‐qPCR: reverse transcription quantitative PCR
SNpc: substantia nigra pars compacta
SOD: superoxide dismutase
TH: tyrosine hydroxylase
TUNEL: terminal deoxynucleotidyl transferase‐mediated dUTP nick end‐labeling
Ψm: mitochondrial membrane potential

## 1 Introduction

Parkinson's disease (PD) is the most common neurodegenerative disorder after Alzheimer's disease, mainly affecting the people above the age of 65 [1, 2]. The main clinical manifestations are bradykinesia, rigidity, resting tremor, and postural imbalance [3]. The typical pathological characteristics of PD are the depletion of dopaminergic neurons in the substantia nigra pars compacta (SNpc) and the formation of Lewy bodies including α‐synuclein [1, 4, 5]. Currently, the mainstay of PD management is symptomatic therapy with drugs that elevate dopamine content or directly activate dopamine receptors [4], but it can only attenuate some of the clinical symptoms. Thus, novel therapeutic approaches are urgently required.

Insulin‐like growth factor 1 (IGF‐1) is a neurotrophic factor that is highly expressed in the brain, especially in neuron‐rich regions [6, 7]. Increasing evidence demonstrated the neuroprotective function of IGF‐1 in PD [8, 9, 10, 11, 12, 13]. Previous studies have reported that IGF‐1 has high affinity for IGFBPs and plays an important role in neuronal growth and repair via interacting with IGF‐binding proteins (IGFBPs) [14, 15, 16]. With respect to IGFBPs, IGFBP2 expression in cerebrospinal fluid has been reported to be positively correlated with the biomarkers of Alzheimer's disease, a neurodegenerative disease [17]. IGFBP2 could also contribute to the neurodegeneration of Alzheimer's disease [17, 18]. Mashayekhi et al. observed the increases in IGF‐1 and IGFBP2 concentrations in serum and cerebrospinal fluid specimens from PD patients, further suggesting that IGFBP2 may be involved in the progression and pathogenesis of PD [19]. However, the knowledge of how IGFBP2 affects PD pathogenesis has not been fully clarified. In this study, we aimed to explore the function of IGFBP2 in PD and its underlying molecular mechanisms in vivo and in vitro.

## 2 Materials and Methods

### 2.1 Animals, Experimental Design, and Dosing

Healthy male Wistar rats, weighing 280 ± 20 g and aged 10–12 weeks old, were housed under a 12 h light/dark cycle and a controlled laboratory environment with free access to water and food. A total of 108 rats were used in our study. All procedures were approved by the Committee on the Care and Use of Animals of Shengjing Hospital of China Medical University (Ethics Approval Number: 2022PS1126K; approval date: 24 November 2022).

For mRNA sequencing (mRNA‐seq), a total of 6 rats were randomly divided into 2 groups, 3 rats in Control group, and 3 rats in PD group. To prevent adrenergic neuron damage caused by 6‐OHDA, the rats were given 20 mg/kg imipramine of intraperitoneal injection 30 min prior to modeling. The in vivo model of PD was established by the unilateral 6‐OHDA lesion as described previously. In brief, the anesthetized rats were fixed on a stereotactic frame and stereotactically injected with 6‐OHDA (20 μg in 3 μL of 2% ascorbic acid) unilaterally into the medial forebrain bundle (MFB) [20]. Three weeks after 6‐OHDA infusion, the rats were euthanized by carbon dioxide asphyxiation, and the whole brain tissues including SNpc were harvested. The brain was flash‐frozen in liquid nitrogen, and then the midbrain was dissected. The SNpc was located under the stereomicroscope according to the rat brain stereotaxic atlas. SNpc samples were collected using a syringe needle and microsurgery forceps.

To investigate the function of IGFBP2 in PD in vivo, the rats were randomly distributed into three groups: Control (36 rats), 6‐OHDA (36 rats), rIGFBP2 + 6‐OHDA (30 rats). Recombinant IGFBP2 protein (rIGFBP2) was purchased from Cloud‐Clone Corp (Wuhan, China). The rats in the rIGFBP2 + 6‐OHDA group were injected with rIGFBP2 (50 μg/3 μL PBS) by a single intra‐MFB injection 6 h before 6‐OHDA infusion. Control rats were injected with the equal solvent. Experimental design was displayed in Figure 2A. According to Narbute et al.'s method [20], 6‐OHDA or rIGFBP2 was injected into the intra‐MFB using the following coordinates: −2.2 mm anteroposterior, +1.5 mm mediolateral, and −8.0 mm dorsoventral relative to the bregma.

### 2.2 RNA Sequencing and Bioinformatics Analysis

Total RNA was extracted from six tissues samples, and the integrities were evaluated by Agilent 2100. After RNA‐seq libraries were prepared, RNA sequencing was performed using illumine's NovaSeq 6000 sequencing platform. Raw data were filtered using Fastp (https://github.com/OpenGene/fastp) to generate clean data. The rat reference genome (Rnor6.0 version) and annotation files were downloaded from Ensembl database (http://www.ensembl.org/index.html). The gene‐level quantification was performed by HTSeq (version 0.6.1). DESeq2 software (version 1.24.0) was used for differential expression analysis between the two comparison combinations. The GeneCards database (http://www.genecards.org) was used for obtaining the Parkinson‐related genes. Venn diagram was drawn using an online Venn diagram tool (https://csbg.cnb.csic.es/BioinfoGP/venny.html).

### 2.3 Rotational Behavior

To assess the severity of 6‐OHDA‐caused lesions, the rats underwent rotational behavior testing at 2 and 3 weeks after 6‐OHDA infusion. As previously described [21, 22, 23], the rats were injected subcutaneously with 0.5 mg/kg apomorphine, and then placed in a beaker with a 18.1‐cm diameter and 5000 mL capacity. The rotations were monitored for a period of 30 min.

### 2.4 Catalepsy Test

Catalepsy was evaluated using the bar test as described earlier [24]. The forepaws of rats were placed on a horizontal 9 cm high bar with the hind quarters on a platform. Time was recorded when both forepaws were removed from the bar or when the animals climbed onto the bar with all limbs. A testing cut‐off time of 120 s was applied.

### 2.5 Pole Test

The Pole test was performed following the previous method [24]. A prepared wooden pole (55 cm in height and 1 cm in diameter) was placed vertically in the center of the cage. Rats were placed head up near the top of the pole. The time to turn its head down (known as time to turn, *t*‐turn) and the total time (*t*‐total) to move down until the animals climbed down the pole (including adjusting itself downward) were recorded.

### 2.6 Immunohistochemistry

After the behavioral tests, the rats (*n* = 3 per group for mRNA‐seq; *n* = 6 per group for further animal experiments) were euthanized and SNpc samples were prepared, followed by soaking in 4% paraformaldehyde. For tyrosine hydroxylase (TH) immunohistochemistry (IHC), the experimental procedures were as reported by Yan et al. [25]. In brief, the fixed samples were paraffin embedded and sectioned at 5‐μm thickness. After dewaxing and rehydrating, the antigen retrieval was performed. Then, the sections were incubated with 3% H_2_O_2_ for 15 min and blocked with 1% BSA for 15 min at the room temperature. The sections were incubated overnight with a primary anti‐TH antibody (1:200; ab112, Abcam). After being washed, they were incubated with secondary antibody (1:500; #31460; ThermoFisher) for 60 min. Immunostaining was visualized using diaminobenzidine (DAB) solution, and counterstaining was performed with hematoxylin. Images of stained sections were obtained under a 40× or 200× microscope.

### 2.7 Cell Culture and Treatment

Rattus norvegicus pheochromocytoma PC12 cells were purchased from Procell Life Science&Technology Co., Ltd. (Wuhan, China) and cultured in RPMI‐1640 cell culture medium with 95% air and 5% CO_2_ at room temperature. For induction of neuronal phenotype, PC12 cells were treated with 100 ng/mL nerve growth factor (NGF), as reported previously [26].

All experiments were conducted in differentiated PC12 cells (below passage 25). The differentiated PC12 cells (4 × 10^3^ cells/well) were placed on a 96‐well dish. To induce PD model in vitro, the cells were treated with 50 μM 6‐OHDA for 24 h. For rIGFBP2 and recombinant IGF‐1 protein (rIGF‐1) treatment, PC12 cells were co‐treated with 50 ng/mL rIGFBP2 and 50 ng/mL rIGF‐1 for 24 h, respectively, followed by treatment with 50 μM 6‐OHDA.

### 2.8 Cell Counting Kit‐8 Assay

Cell viability was examined using a cell counting kit‐8 (CCK‐8) assay. Briefly, 10 μL of CCK‐8 reagent (Beyotime, Shanghai, China) was added to the well, and the cells were incubated in an incubator for 2 h with 5% CO_2_ at 37°C. The optical densities of each well were read on a microplate reader at 450 nm.

### 2.9 Biochemistry Analysis

Reactive oxygen species (ROS), malondialdehyde (MDA), and superoxide dismutase (SOD) detection kits were all purchased from Nanjing Jiancheng Institute of Biotechnology (Nanjing, China). Glutathione peroxidase (GSH‐Px) determination kit was obtained from Wanleibio (Shengyang, China). ROS, MDA levels and SOD, GSH‐Px activities in SNpc tissues and cultured PC12 cells were determined using the commercially available kits as per manufacturer protocols. Adenosine triphosphate (ATP) production in tissue samples was determined using ATP detection kit (Beyotime, Shanghai, China). LDH level in cell supernatant was measured using LDH detection kit (Jiancheng Bio) according to manufacturer's instruction.

### 2.10 Reverse Transcription Quantitative PCR (RT‐qPCR) Assay

Total RNA was isolated using TRIpure reagent (BioTeke, Beijing, China). RT‐qPCR template, first‐stranded cDNA, was generated using BeyoRT II M‐MLV reverse transcriptase (Beyotime). Then, IGFBP2 expression level was assessed by RT‐qPCR using SYBR Green PCR Master Mix (Solarbio, Beijing, China) in accordance with the manufacturer's instruction. The primers for IGFBP2 were synthesized from GenScript (Nanjing, China). The following sequences were used: forward 5′‐TTGCGGCGTCTACATCC‐3′ and reverse 5′‐TCCCTCCGAGTGGTCATC‐3′. The mRNA expression level was normalized using β‐actin, and relative mRNA expression was calculated using the $2^{-\Delta \Delta C_{t}}$ method [27].

### 2.11 Western Blotting Analysis

Total proteins from SNpc tissues and PC12 cells were extracted by RIPA lysis buffer (Beyotime) and PMSF. Mitochondria proteins was extracted using the Cytoplasmic and Mitochondria protein isolation kit (BOSTER, Wuhan, China) according to the protocol of manufacturer. A BCA protein assay kit (Beyotime) was applied to determine the protein concentration. Western blotting assay was performed based on 15–30 μg of protein separated by SDS‐PAGE, and the proteins were transferred onto PVDF membranes (Thermo Fisher Scientific, USA). After being blocked with western blotting sealing fluid (Proteintech, China) for 1 h, the membranes were incubated overnight with the following primary antibodies at 4°C, namely, anti‐IGFBP2 (1:500; Abclonal); anti‐α‐synuclein (1:1000; Abclonal); anti‐Cytochrome *c* (1:1000; Abclonal); anti‐phospho (p)‐IGF‐1R^Tyr1131^ (1:2000; Affinity); anti‐IGF‐1R (1:1000; Affinity); anti‐phospho (p)‐AKT^Ser473^ (1:2000; Affinity); anti‐AKT (1:1000; Affinity); anti‐Bcl‐2 (1:1000; Affinity); anti‐Bax (1:1000; Cell Signaling Technology); anti‐cleaved caspase‐9 (1:1000; Cell Signaling Technology); anti‐cleaved caspase‐3 (1:1000; Cell Signaling Technology). After being washed 4 times with TBST, the membranes were incubated for 40 min with anti‐rabbit HRP‐connected secondary antibody (1:10,000) at 37°C. The antibody‐bound proteins were visualized using enhanced chemiluminescence (ECL) reagent (Proteintech) and analyzed using Gel‐Pro‐Analyzer software.

### 2.12 JC‐1 Staining

Determination of the mitochondrial membrane potential (Ψm) was performed by an assay kit with JC‐1 (Beyotime). According to the protocols of the manufacturer, the cells were stained with JC‐1 staining buffer (5×) for 20 min, washed twice with 1× buffer, and then the fluorescence intensity was measured using Image‐Pro Plus software (Version 6.0).

### 2.13 TUNEL Staining

Terminal deoxynucleotidyl transferase‐mediated dUTP nick end‐labeling (TUNEL) assay was carried out using an in situ cell death detection kit (Roche, Switzerland) as the manufacturer's protocol. Briefly, the fixed SNpc tissues were cut into 5 μm thick and the sections were subjected to paraffin embedding, deparaffinization, and rehydration, and then permeabilized with 0.1% Triton X–100 (Beyotime). The sections were then incubated for 60 min with 50 μL of TUNEL reaction mixture at 37°C. After nuclear staining with DAPI (Aladdin, Shanghai, China), the apoptotic cells were visualized using a fluorescent microscope (200×).

### 2.14 Hoechst 33258 Staining

Cultured PC12 cells were stained with Hoechest staining solution for 5 min in accordance with the protocol of the kit (Beyotime). After washing twice with PBS, the apoptotic cells were identified and counted under a fluorescent microscope (200×).

### 2.15 Statistical Analysis

All experiments were conducted at least three times. All statistical analysis including normality tests were performed by GraphPad Prism software (version 8.0) and presented as mean ± standard deviation (SD). Before statistical analysis, data were checked for outliers and normality using Shapiro–Wilk test. Data that do not exhibit a normal/Gaussian distribution were analyzed via a nonparametric equivalent. Pairwise differences between groups were indicated by Student's *t*‐test, and comparison analysis among multiple groups was tested by one‐way analysis of variance (ANOVA) followed by Tukey's multiple comparisons test. Results were considered as statistically significant when *p* value was less than 0.05.

## 3 Results

### 3.1 Establishment of PD Rat Model and Analysis of mRNA Sequencing

**FIGURE 1:**
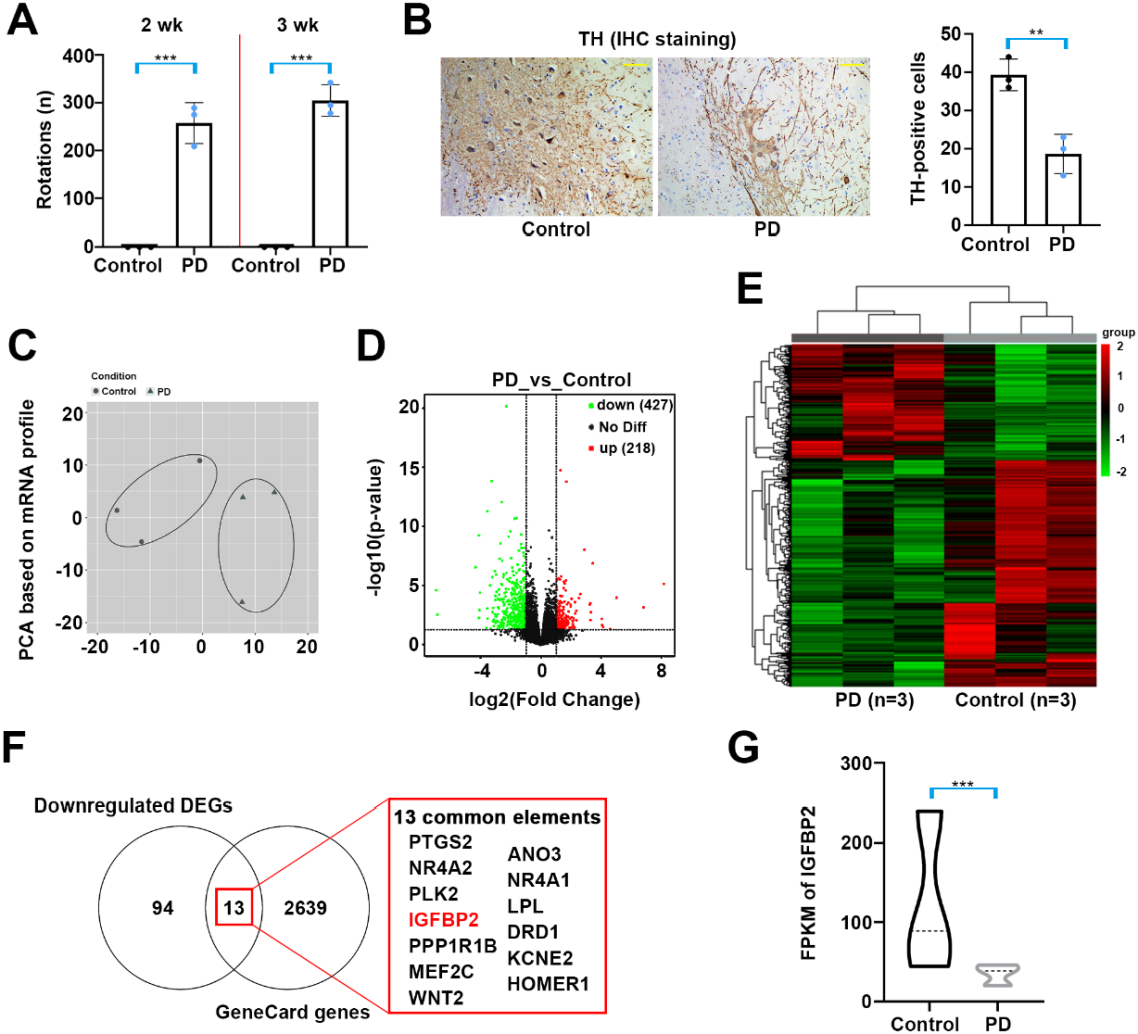
Differential expression of mRNAs in PD rats. (A) The rats were injected with 6‐OHDA to induce the PD animal model. Apomorphine‐induced rotational behavior was tested at 2 and 3 weeks after modeling. (B) IHC staining of TH in OHDA‐lesioned rats (3 weeks). Bar = 100 µm. (C) Bidimensional principal component analysis (PCA) showing distinct clustering of gene profiles in 6‐OHDA‐induced PD rats and control rats. (D, E) Volcano plot and heatmap showing differential expression of mRNAs (|log_2_ fold change (FC)| > 1 and *p* < 0.05). (F) Venn graph depicting the common genes of downregulated differentially expressed genes (DEGs, log_2_FC < −1.5 and *p* < 0.001) and parkinson‐related genes from the GeneCards database. (G) The FPKM value of IGFBP2 was shown. Group sizes were: *N* = 3 animals per group. **p < 0.01, ***p < 0.001. * = control vs. 6‐OHDA.

As shown in Figure 1A, the rotational behavior was assessed at 2 and 3 weeks after modeling. It was found that apomorphine induced abnormal contralateral rotations in PD rats, as the PD‐like syndrome developed. There was obvious reduction in the number of TH‐positive cells in the SNpc tissues of 6‐OHDA‐induced rats (Figure 1B). These data indicated a successful establishment of the PD model in rats. To identify differentially expressed mRNAs in PD, the area containing SNpc was collected and NovaSeq 6000 sequencing platform was applied. As shown in Figure 1C, principal component analysis (PCA) revealed distinct sample clustering between groups. A volcano plot and heatmap showed significantly differential expression patterns between PD and control group, and the screening criterion was set as |log_2_ fold change (FC)| > 1 and *p* < 0.05 (Figure 1D,E). In total, 645 differentially expressed genes (DEGs) were found, of which 218 DEGs were upregulated and 427 DEGs were downregulated. Besides, to narrow the screening, the 107 DEGs was obtained with log_2_FC < −1.5 and *p* < 0.001 as the criteria. Using the GeneCards database, 2652 genes were obtained. Venn analysis for screening the common active genes that related to PD. According to the literature search, the DEG named IGFBP2 was scrutinized (Figure 1F). FPKM represents the expression quantity of the gene [28]. Figure 1G shows that IGFBP2 expression was downregulated in the SNpc tissues of PD rats 3 weeks after 6‐OHDA injection.

### 3.2 rIGFBP2 Treatment Aggravates Behavioral Deficits of PD Model Rats

**FIGURE 2:**
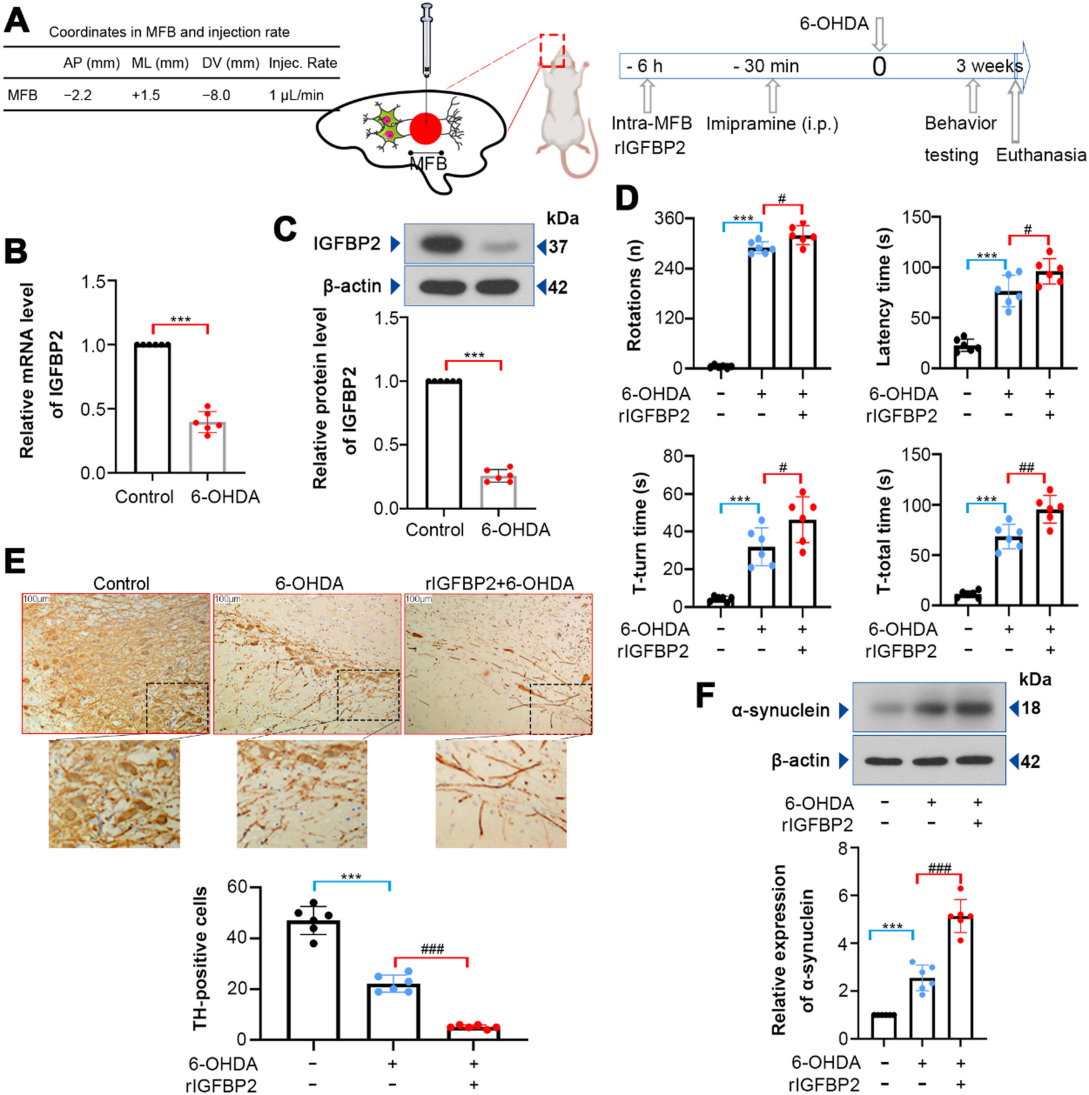
Effect of IGFBP2 on PD‐like phenotypes induced by 6‐OHDA in rats. (A) Schematic representation showing the experiment process of this study. (B, C) RT‐qPCR and western blotting analysis revealed that IGFBP2 expression was downregulated in the substantia nigra pars compacta (SNpc) of 6‐OHDA‐induced PD rats. (D) Behavioral tests showing the effect of IGFBP2 on 6‐OHDA‐induced behavioral impairment (Rotation, *F* (2, 15) = 745.7; Latency time, *F* (2, 15) = 59.48; *T*‐turn time, *F* (2, 15) = 33.01; *T*‐total time, *F* (2, 15) = 95.94). (E) Representative images showed IHC staining of TH in the SNpc of 6‐OHDA‐lesioned rats, followed by quantification of the number of TH‐positive cells (*F* (2, 15) = 188.2). (F) Western blotting analysis depicting α‐synuclein expression in the SNpc tissues (*F* (2, 15) = 102.3). Data were expressed as mean ± SD. Group sizes were: *N* = 6 animals per group. ***p < 0.001, ^#^ *p* < 0.05, ^##^ *p* < 0.01, ^###^ *p* < 0.001. * = control versus 6‐OHDA, ^#^ = 6‐OHDA versus rIGFBP2 + 6‐OHDA.

To investigate the role of IGFBP2 in PD, the rats were injected with rIGFBP2 protein, followed by 6‐OHDA infusion (Figure 2A). As displayed in Figure 2B,C, IGFBP2 expression at mRNA and protein levels was downregulated in the SNpc tissues of PD rats (*p* < 0.001). The 6‐OHDA‐lesioned rats displayed increased number of rotations (contralateral to the lesion), and rIGFBP2 protein treatment resulted in the increase in rotational behavior (*p* = 0.0122). The rIGFBP2‐treated rats showed severe catalepsy as compared to the PD group (*p* = 0.0340). We also noted that the rats treated with rIGFBP2 took more time to turn and descend down the pole than the PD rats (Figure 2D; *T*‐turn time, *p* = 0.0390; *T*‐total time, *p* = 0.0016). TH deficiency is linked to the pathogenesis of PD [29]. Immumohistochemical staining revealed that 6‐OHDA treatment significantly reduced the number of TH‐positive cells, while rIGFBP2 enhanced this change (Figure 2E; *p* < 0.001). Western blotting analysis showed that rIGFBP2 protein elevated α‐synuclein expression in the SNpc tissues of PD rats (Figure 2F; *p* < 0.001). Collectively, rIGFBP2 protein may exacerbate 6‐OHDA‐lesioned neurobehavioral deficits.

### 3.3 rIGFBP2 Administration Increases 6‐OHDA‐Induced Oxidative Stress, Mitochondrial Dysfunction, and Apoptosis

**FIGURE 3:**
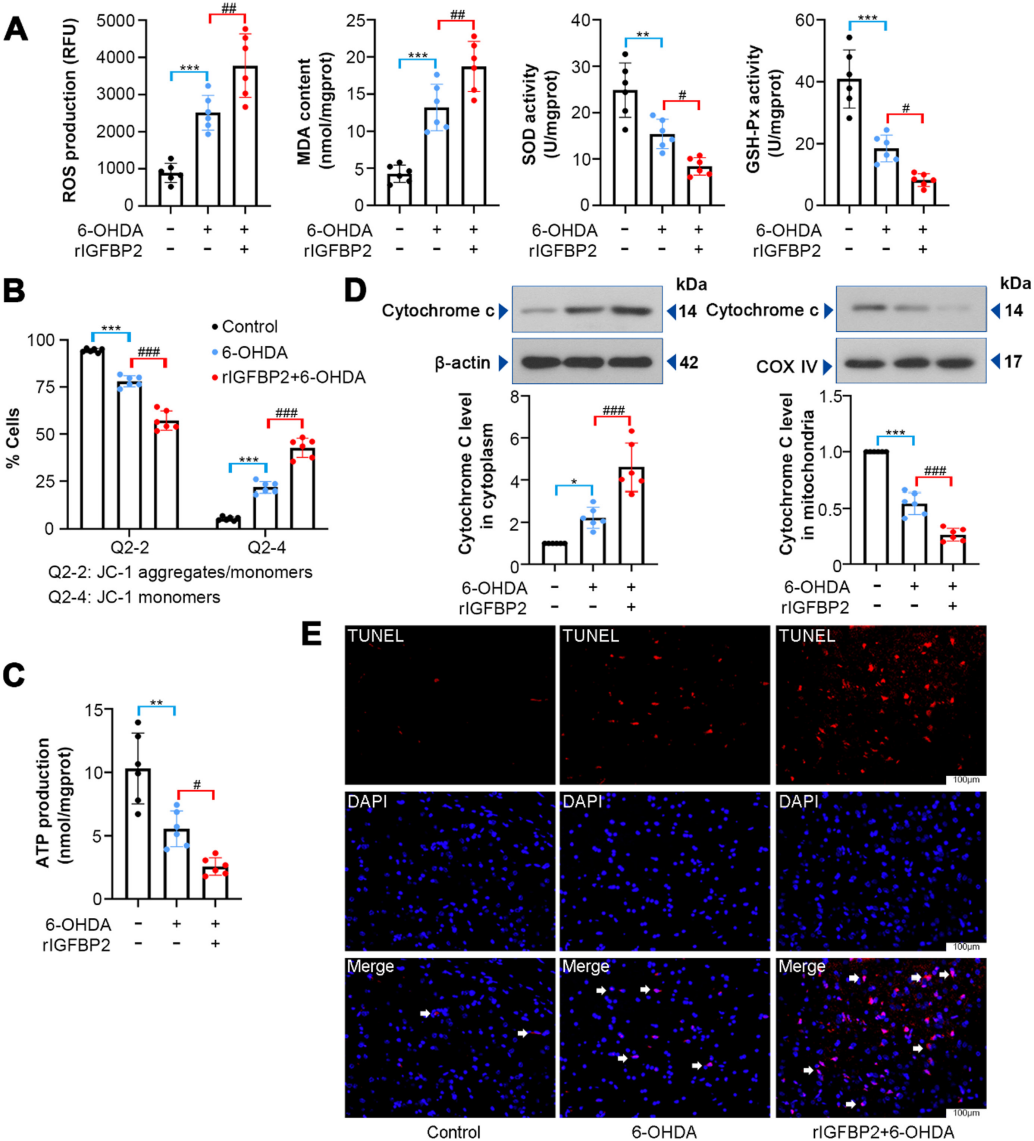
Effect of IGFBP2 on 6‐OHDA‐induced oxidative stress and mitochondrial impairment in rats. (A) ROS production, MDA content, and SOD and GSH‐Px activities in the SNpc were tested using corresponding commercial assay kits (ROS, *F* (2, 15) = 37; MDA, *F* (2, 15) = 42.56; SOD, *F* (2, 15) = 25.42; GSH‐Px, *F* (2, 15) = 45.15). (B) JC‐1 staining indicating the changes in mitochondrial membrane potential (Ψm; Q2‐2, *F* (2, 15) = 175.4; Q2‐4, *F* (2, 15) = 175.5). (C) ATP level was assessed by ATP detection kit (*F* (2, 15) = 26.63). (D) Western blotting analysis revealing cytochrome *c* expression in cytoplasm and mitochondria in the SNpc of rats (cytochrome *c* in cytoplasm, *F* (2, 15) = 38.49; cytochrome *c* in mitochondria, *F* (2, 15) = 193.7). (E) Representative images of TUNEL‐positive staining in the SNpc tissues. Data were expressed as mean ± SD. Group sizes were: *N* = 6 animals per group. *p < 0.05, **p < 0.01, ***p < 0.001, ^#^ *p* < 0.05, ^##^ *p* < 0.01, ^###^ *p* < 0.001. * = control versus 6‐OHDA, ^#^ = 6‐OHDA versus rIGFBP2 + 6‐OHDA.

Oxidative stress, mitochondrial dysfunction, and apoptosis are associated with the pathological process of neurodegeneration [30]. To determine whether IGFBP2 impacts oxidative stress and mitochondrial dysfunction in PD, the following assays were applied. As shown in Figure 3A, 6‐OHDA led to a marked increase in ROS production and MDA content, and a decline in SOD and GSH‐Px activities, whereas rIGFBP2 treatment exacerbated these changes (*p* < 0.05). Compared with the 6‐OHDA group, the rIGFBP2 protein reduced Ψm (*p* < 0.001) and ATP production (*p* = 0.0354) in the SNpc tissues (Figure 3B,C). Western blotting analysis showed that rIGFBP2 could increase the release of cytochrome *c* from the mitochondria (Figure 3D,E; *p* < 0.001). TUNEL staining uncovered that rIGFBP2 elevated the number of TUNEL‐positive cells (Figure 3F). These data indicated that the rIGFBP2 treatment increases 6‐OHDA‐induced oxidative stress, mitochondrial dysfunction, and apoptosis.

### 3.4 IGFBP2 Administration Inhibits the IGF‐1R/AKT Signaling Pathway

**FIGURE 4:**
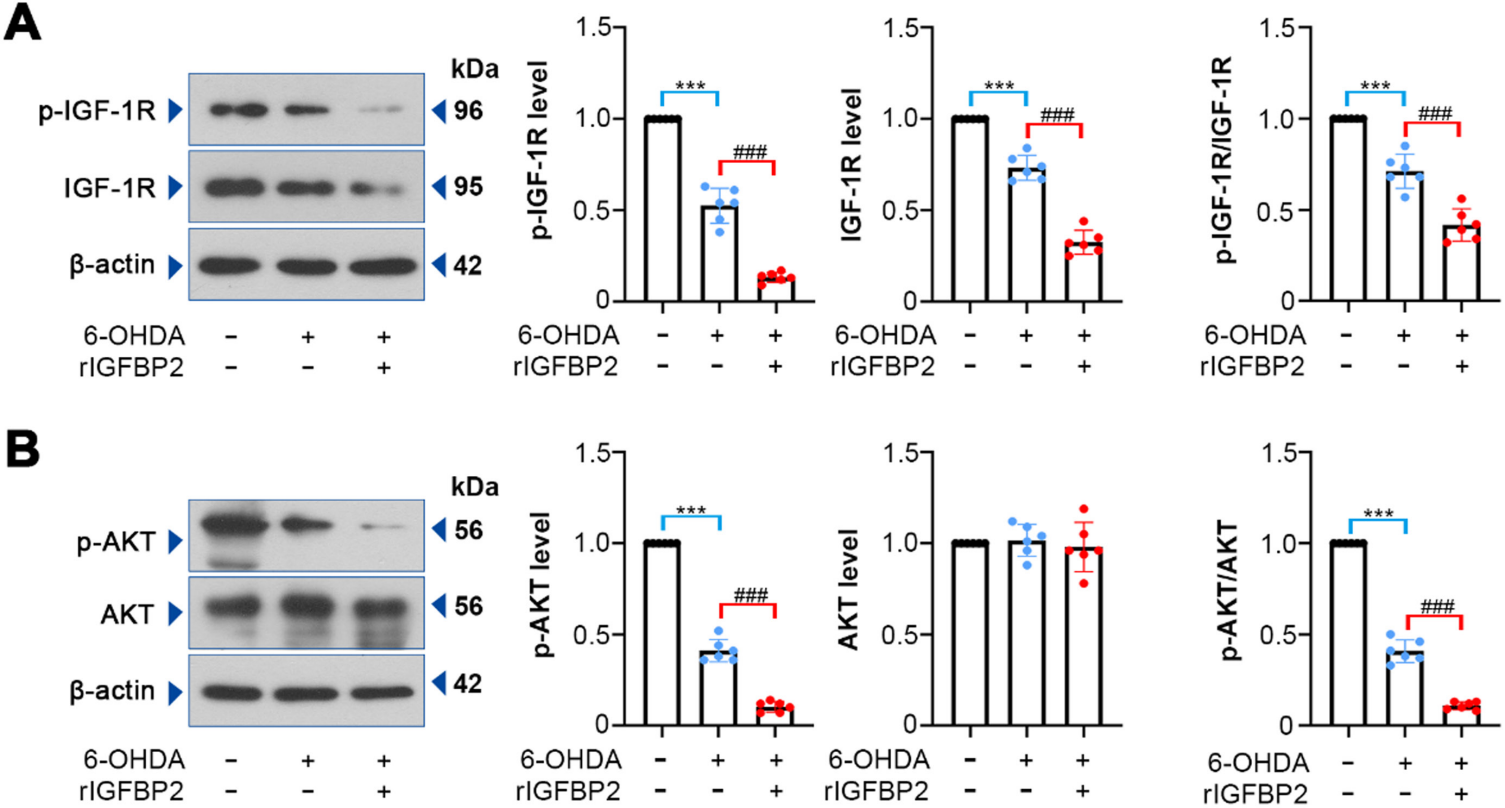
Effect of IGFBP2 on IGF‐1R/AKT signaling pathway. (A, B) Relative expression levels of IGF‐1R, p‐IGF‐1R, AKT, and p‐AKT in the SNpc were measured by western blotting (p‐IGF‐1R, *F* (2, 15) = 345.4; IGF‐1R, *F* (2, 15) = 229.7; p‐IGF‐1R/IGF‐1R, *F* (2, 15) = 92.01; p‐AKT, *F* (2, 15) = 811.5; AKT, *F* (2, 15) = 0.2335; p‐AKT/AKT, *F* (2, 15) = 851.2). Data were expressed as mean ± SD. Group sizes were: *N* = 6 animals per group. ***p < 0.001, ^###^ *p* < 0.001. * = control versus 6‐OHDA, ^#^ = 6‐OHDA versus rIGFBP2 + 6‐OHDA.

The IGF‐1 signaling pathway mediates neuroprotection via regulating oxidative stress, mitochondrial dysfunction, and apoptosis [11, 31, 32]. To determine the underlying mechanism by which IGFBP2 functions, we detected the levels of IGF‐1R/AKT signaling‐related proteins. It was demonstrated that the rIGFBP2 protein resulted in obvious reduction in phosphorylated (p)‐IGF‐1R and IGF‐1R levels in the SNpc tissues of PD rats (Figure 4A; *p* < 0.001). IGFBP2 also declined the phosphorylation of AKT, a downstream target of IGF‐1R (Figure 4B; *p* < 0.001). These results suggest that IGFBP2 inhibits the IGF‐1R/AKT signaling pathway.

### 3.5 Effect of IGFBP2/IGF‐1 on 6‐OHDA‐Induced Damage of PC12 Cells

**FIGURE 5:**
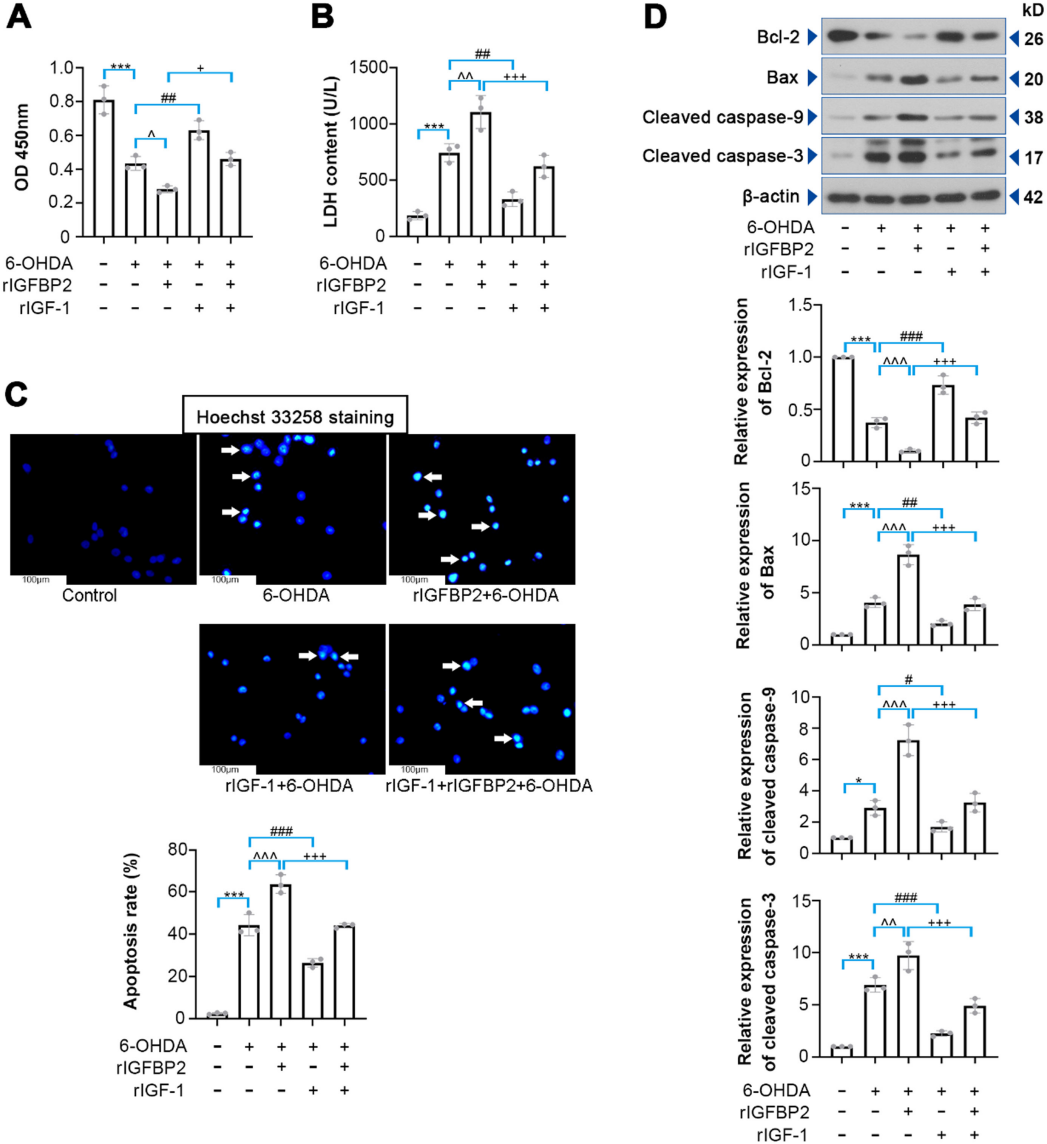
Effect of IGFBP2 on apoptosis induced by 6‐OHDA in differentiated PC12 cells. The differentiated PC12 cells were co‐treated with rIGFBP2 and rIGF‐1 for 24 h, and then subjected to 6‐OHDA for 24 h. (A) CCK‐8 assay was performed to determine cell viability (*F* (4, 10) = 45.19). (B) LDH content was evaluated using a LDH detection kit (*F* (4, 10) = 45.50). (C) Apoptosis induced by 6‐OHDA was determined with Hoechst 33258 staining (*F* (4, 10) = 160.7). (D) Bax, Bcl‐2, cleaved caspase‐9, and cleaved caspase‐3 expression were determined by western blotting in PC12 cells (Bax, *F* (4, 10) = 82.57; Bcl‐2, *F* (4, 10) = 136.6; cleaved caspase‐9, *F* (4, 10) = 53.36; cleaved caspase‐3, *F* (4, 10) = 65.57). Data were expressed as mean ± SD. Group sizes were: *N* = 3 wells per group. *p < 0.05, ***p < 0.001, ^*p* < 0.05, ^^*p* < 0.01, ^^^*p* < 0.001, ^#^ *p* < 0.05, ^##^ *p* < 0.01, ^###^ *p* < 0.001, ^+^ *p* < 0.05, ^+++^ *p* < 0.001. * = control versus 6‐OHDA, ^ = 6‐OHDA versus rIGFBP2 + 6‐OHDA, ^#^ = 6‐OHDA versus rIGF‐1 + 6‐OHDA, ^+^ = rIGFBP2 + 6‐OHDA versus rIGF‐1 + rIGFBP2 + 6‐OHDA.

We further verified the function of IGFBP2 in neuronal PC12 cells. As shown in Figure S1A, the PC12 cells differentiated into neuron‐like cells after NGF induction. CCK‐8 assay showed that 6‐OHDA (50 μM) led to a significant reduction in cell viability as compared to the control group (Figure S1B; *p* < 0.01). To determine the role of IGFBP2/IGF‐1 in 6‐OHDA‐treated PC12 cells, the cells were co‐treated with rIGFBP2 and rIGF for 24 h, followed by 6‐OHDA induction. It was found that rIGFBP2 administration reduced cell viability in the presence of 6‐OHDA (*p* = 0.0317), whereas rIGF‐1 partially attenuated this change (Figure 5A; *p* = 0.0124). Treatment of rIGFBP2 elevated LDH content (*p* = 0.0052), which was diminished by rIGF‐1 (Figure 5B; *p* < 0.001). Hoechst 33258 staining analysis showed that rIGFBP2 increased cell apoptosis (*p* < 0.001), while rIGF‐1 improved it (Figure 5C; *p* < 0.001). Relative expression of proteins associated with apoptosis was measured using western blotting assays, and the results suggested that rIGFBP2 treatment decreased Bcl‐2 level, but increased Bax, cleaved caspase‐9, and cleaved caspase‐3. However, IGF‐1 partially reversed these changes (Figure 5D; *p* < 0.001).

### 3.6 Effect of IGFBP2/IGF‐1 on 6‐OHDA‐Induced Oxidative Stress and Mitochondrial Dysfunction in PC12 Cells

**FIGURE 6:**
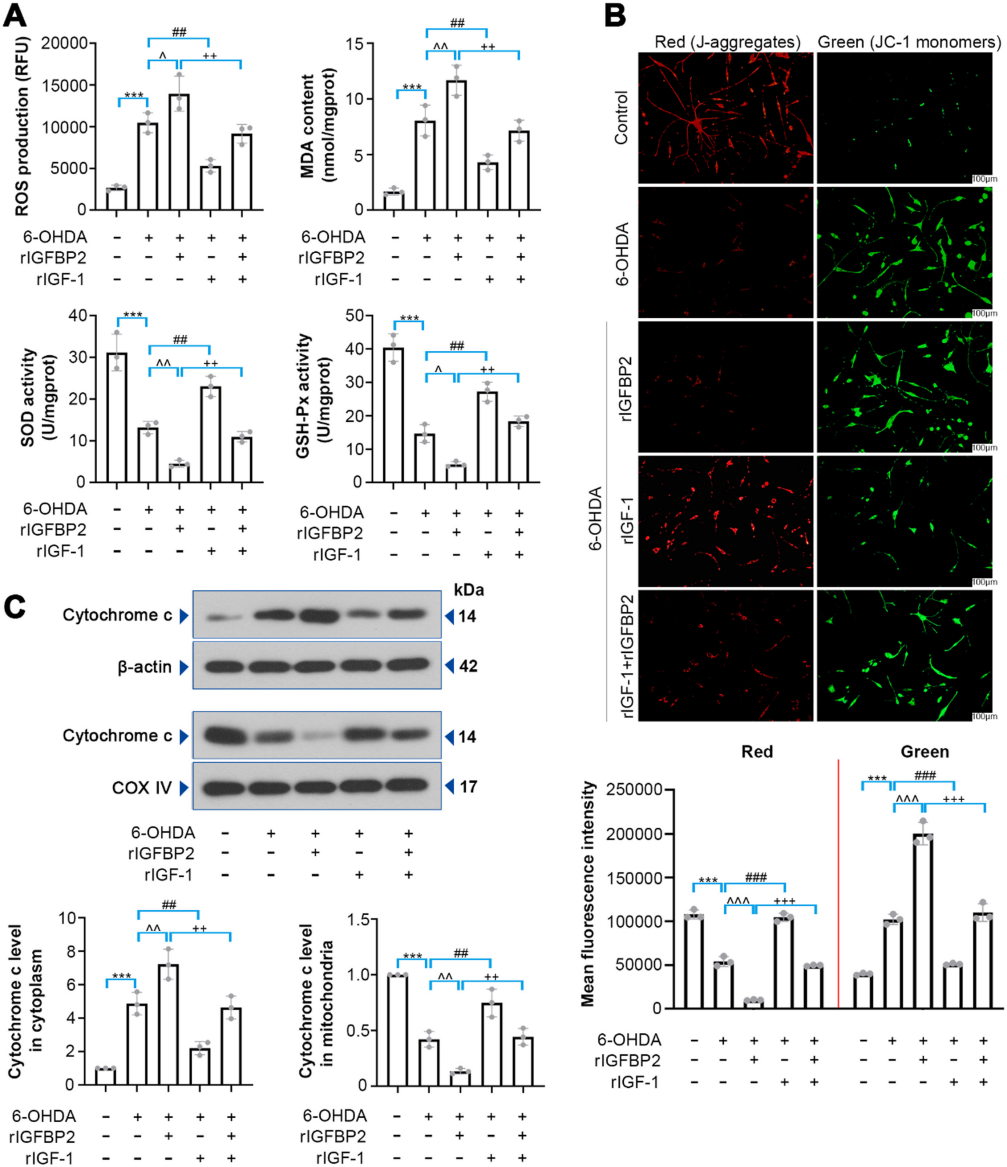
Effect of IGFBP2 on oxidative stress and mitochondrial function in 6‐OHDA‐lesioned PC12 cells. (A) ROS and MDA levels, SOD and GSH‐Px activities were determined by the commercial assay kits (ROS, *F* (4, 10) = 37.55; MDA, *F* (4, 10) = 41.91; SOD, *F* (4, 10) = 55.79; GSH‐Px, *F* (4, 10) = 75.03). (B) Changes in Ψm were evaluated by JC‐1 staining. (C) Relative expression of Cytochrome *c* in cytoplasm and mitochondria was detected by western blotting analysis (cytochrome *c* in cytoplasm, *F* (4, 10) = 47.39; cytochrome *c* in mitochondria, *F* (4, 10) = 62). Data were expressed as mean ± SD. Group sizes were: *N* = 3 wells per group. ***p < 0.001, ^*p* < 0.05, ^^*p* < 0.01, ^^^*p* < 0.001, ^##^ *p* < 0.01, ^###^ *p* < 0.001, ^++^ *p* < 0.01, ^+++^ *p* < 0.001. * = control versus 6‐OHDA, ^ = 6‐OHDA versus rIGFBP2 + 6‐OHDA, ^#^ = 6‐OHDA versus rIGF‐1 + 6‐OHDA, ^+^ = rIGFBP2 + 6‐OHDA versus rIGF‐1 + rIGFBP2 + 6‐OHDA.

The role of IGFBP2/IGF‐1 in 6‐OHDA‐induced oxidative stress and mitochondrial dysfunction was further investigated. As illustrated in Figure 6A, rIGFBP2 treatment elevated ROS generation, MDA content, and declined SOD and GSH‐Px activities in 6‐OHDA‐induced PC12 cells, which were partially reversed by rIGF‐1 (*p* < 0.01). Besides, rIGFBP2 treatment decreased Ψm (*p* < 0.001), and rIGF‐1 increased it (Figure 6B; *p* < 0.001). The effect of rIGFBP2 on promoting cytochrome *c* release from the mitochondria was weakened following rIGF‐1 treatment (Figure 6C; *p* < 0.01). These results suggest that IGFBP2 may aggravate 6‐OHDA‐induced oxidative stress and mitochondrial dysfunction via negatively regulating IGF‐1.

## 4 Discussion

In this work, we identified differentially expressed mRNAs involved in PD and highlighted the function of IGFBP2 in PD development. Treatment with rIGFBP2 protein aggravated 6‐OHDA‐caused neurobehavioral defects in rats. rIGFBP2 protein caused excessive oxidative stress, mitochondrial dysfunction, apoptosis, and IGF‐1R/AKT inactivation. In 6‐OHDA‐treated PC12 cells, rIGFBP2 treatment resulted in decreased cell viability, increased apoptosis, oxidative stress, and mitochondrial dysfunction, which were partially reversed by rIGF‐1. Our findings provide new evidence that IGFBP2 may contribute to 6‐OHDA‐induced apoptosis, oxidative stress, and mitochondrial dysfunction in PD via regulating the IGF‐1R/AKT signaling pathway.

IGFBP2, a member of the six IGF‐binding proteins, has been reported to be involved in the development of various human diseases including neurodegenerative disease [33]. IGFBP2 is locally expressed in the central nervous system. The reduced expression of IGFBP2 in the cortex of mice with Alzheimer's disease is found, and IGFBP2 may be linked to metabolic dysregulation and neurodegeneration [34]. Wang et al. found that IGFBP2 expression is reduced in the brain tissue of ischemia‐injured rats [35]. Our mRNA‐seq and RT‐qPCR analysis demonstrated the downregulated IGFBP2 expression in the SNpc tissues of PD rats, which was consistent with previous studies. Lane et al. [18], Bonham et al. [34] and Quesnel et al. [17] have illustrated that IGFBP2, the most abundant IGFBPs, may be associated with biomarkers of Alzheimer's disease and contribute to neurodegeneration. Zhang et al. [36] found that the reduction of IGFBP2 can inhibit neuroninflammation in the spinal cords of experimental autoimmune encephalomyelitis. We then evaluate the effect of IGFBP2 in PD. In this study, considering that IGFBP2 is a secreted protein, we applied the rIGFBP2 protein. rIGFBP2 treatment resulted in severe neurological defects, indicating IGFBP2 might exhibit a pathogenic role in PD. Deletion of TH neurons and aggregation of α‐synuclein in SNpc are important characteristics of PD pathogenesis [37, 38]. We noted that TH‐positive neurons were declined and α‐synuclein expression was elevated in PD rats, whereas IGFBP2 enhanced these changes. Our data confirmed that rIGFBP2 aggravates neurodegeneration in PD. Notably, previous study has revealed a reason that may contribute to IGFBP2 downregulation in brain tissue during cerebral injury: more IGFBP2 is transported out of the brain tissue to bind to IGF‐1, owing to the incremental requirement of IGF‐1 of injured brain tissue [35]. IGFBP2 expression may fluctuate due to compensatory mechanism as reported by Wood et al. [39] and Hoeflich et al. [40]. It is possible that IGFBP2 downregulation in SNpc of PD rats happens for the same reason, which requires extended studies in the near future.

Growing evidence suggests that excessive oxidative stress and mitochondrial dysfunction, causing apoptosis of neurons, are responsible for neurodegeneration in PD [30, 41, 42]. The neurotoxic effect of 6‐OHDA is attributed to the production of oxidative stress to generate reactive species, including superoxide radical and hydrogen peroxide [43, 44]. Previous evidence has uncovered an obvious increase in oxidative stress in the SNpc tissues of 6‐OHDA‐treated animals [45]. Reduction of oxidative stress is capable of attenuating 6‐OHDA‐induced neuronal damage [46]. In this work, 6‐OHDA caused a significant elevation in ROS production and MDA content, a decrease in SOD and GSH‐Px activities, indicating that the mechanism of 6‐OHDA toxicity includes the increase in oxidative stress, as mentioned in previous studies. Interestingly, rIGFBP2 treatment enhanced 6‐OHDA‐induced oxidative stress in PD, suggesting that the role of IGFBP2 in PD is associated with increased oxidative stress. Chiu et al. found that reducing IGFBP2 in neurons has anti‐oxidative stress and neuroprotective effects [47]. Moreover, 6‐OHDA can induce neurotoxicity via modulating mitochondrial function [43]. We found that rIGFBP2 reduced ATP level in 6‐OHDA‐lesioned rats. Decreasing ATP generation is a consequence of mitochondrial dysfunction [48]. The release of cytochrome *c* is closely associated with damaged mitochondrial dysfunction [49]. rIGFBP2 administration increased cytochrome *c* release from mitochondria. These results suggest that rIGFBP2 protein aggravates mitochondrial dysfunction in PD. Furthermore, neuronal loss is a key event of PD pathogenesis [43, 50]. We observed enhanced TUNEL‐positive staining in the SNpc of PD rats and increased Hoechst 33258‐stained cells, which was supported by the previous reports [51, 52]. rIGFBP2 protein increased the number of apoptotic cells, along with the increase in pro‐apoptotic proteins Bax, cleaved caspase‐9 and cleaved caspase‐3 and the decrease in anti‐apoptotic Bcl‐2. Hence, the present study suggests that IGFBP2's role in promoting PD development is associated with enhanced oxidative stress, apoptosis, and impaired mitochondrial function.

The underlying mechanism involved in IGFBP2 is further investigated. Recent studies indicated that IGF‐1 system is associated with the development of PD. IGF‐1 can prevent the damage to dopamine neurons in a PD model induced by 6‐OHDA or MPTP [11, 53]. The effect of IGF‐1 is mainly mediated by the IGF‐1R, we thus evaluated the role of IGFBP2 in IGF‐1R activation. Our study in line with a previous research [54] demonstrated that IGF‐1R and p‐IGF‐1R levels were downregulated by 6‐OHDA, and rIGFBP2 treatment further inhibited the IGF‐1‐mediated activation of IGF‐1R in PD. AKT, a downstream target of the IGF‐1 signaling pathway, is considered to be a key protein for IGF‐1‐mediated protection in PD. Activated AKT contributes to the neuroprotection against PD [53, 54]. We found that rIGFBP2 protein inhibited AKT activation, which is consistent with the previous finding [55]. Moreover, IGFBP2 has been reported to negatively modulate IGF‐1 activity in injured brain [35]. Whether IGFBP2's function in PD is IGF‐dependent remains unclear. In our work, neuronal PC12 cells were treated with rIGFBP2 and rIGF‐1, followed by 6‐OHDA induction. Interestingly, rIGFBP2 protein induced obvious damage in PC12 cells, reflected by decreased cell viability and increased LDH content. rIGF‐1 treatment seemed to prevent the damage. These results indicate that the pathogenic role of IGFBP2 may depend on IGF‐1.

In summary, our findings reveal that IGFBP2 in PD exacerbates dopaminergic neuron loss and neurobehavioral deficits through triggering cell apoptosis, excessive oxidative stress, and mitochondrial dysfunction. The pathogenic role of IGFBP2 in PD may be associated with the inhibition of IGF‐1‐mediated AKT activation. Our findings provide a new aspect for the therapy of PD.

## Author Contributions

J.F. and J.A. designed the study; J.A., L.W., and H.Y. carried out all experiments. J.F. supervised the experiments. L.W. and Z.B. analyzed the data. J.A. wrote and checked the manuscript. All authors have read and approved the final version of the manuscript.

## Conflicts of Interest

The authors declare no conflicts of interest.

## Supporting information


**Figure S1.** Effect of IGFBP2 on 6‐OHDA‐induced cell viability in NGF‐induced PC12 cells. (A) PC12 cells were incubated with nerve growth factor (NGF) to induce neurite formation. Representative photomicrographs of PC12 cells with or without NGF exposure. (B) Cell viability was determined using CCK‐8 assay after treatment with 6‐OHDA. Data were expressed as mean ± SD. Group sizes were: *n* = 3 wells per group. **p < 0.01. * = control versus 6‐OHDA.

Data S1.

## Data Availability

All data supporting the results of this study are available with the corresponding author.
