# Supplementary material for: Insulin‐Like Growth Factor Binding Protein 2 Drives Neurodegeneration in Parkinson's Disease: Insights From In Vivo and In Vitro Studies
Source: CNS Neurosci Ther. 2024 Oct 16;30(10):e70076. doi: 10.1111/cns.70076 (PMC11480970; doi:10.1111/cns.70076)
Supplement: Supplementary file 1 — Figure S1. Effect of IGFBP2 on 6‐OHDA‐induced cell viability in NGF‐induced PC12 cells. (A) PC12 cells were incubated with nerve growth factor (NGF) to induce neurite formation. Representative photomicrographs of PC12 cells with or without NGF exposure. (B) Cell viability was determined using CCK‐8 assay after treatment with 6‐OHDA. Data were expressed as mean ± SD. Group sizes were: n = 3 wells per group. **p < 0.01. * = control versus 6‐OHDA. [file CNS-30-e70076-s002.docx]

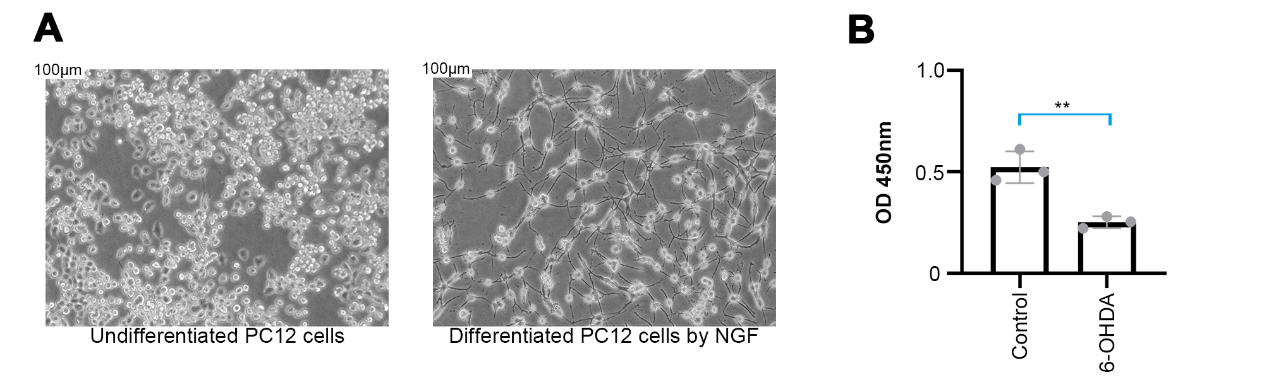


**FIGURE S1** Effect of IGFBP2 on 6-OHDA-induced cell viability in NGF-induced PC12 cells. (A) PC12 cells were incubated with nerve growth factor (NGF) to induce neurite formation. Representative photomicrographs of PC12 cells with or without NGF exposure. (B) Cell viability was determined using CCK-8 assay after treatment with 6-OHDA. Data were expressed as mean ± SD. Group sizes were: n = 3 wells per group. **p<0.01. * = control versus 6-OHDA.
