# Supplementary figures and images for: Insulin‐Like Growth Factor Binding Protein 2 Drives Neurodegeneration in Parkinson's Disease: Insights From In Vivo and In Vitro Studies
Source: CNS Neurosci Ther. 2024 Oct 16;30(10):e70076. doi: 10.1111/cns.70076 (PMC11480970; doi:10.1111/cns.70076)

Figure 2C

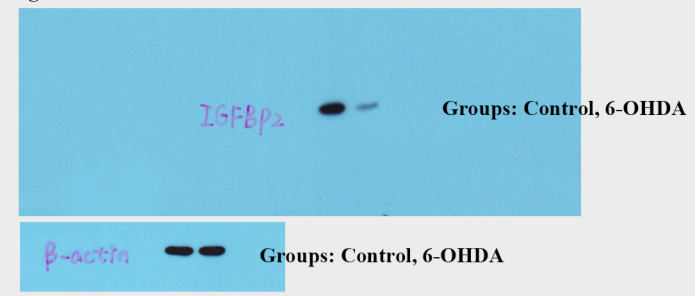

Figure 2F

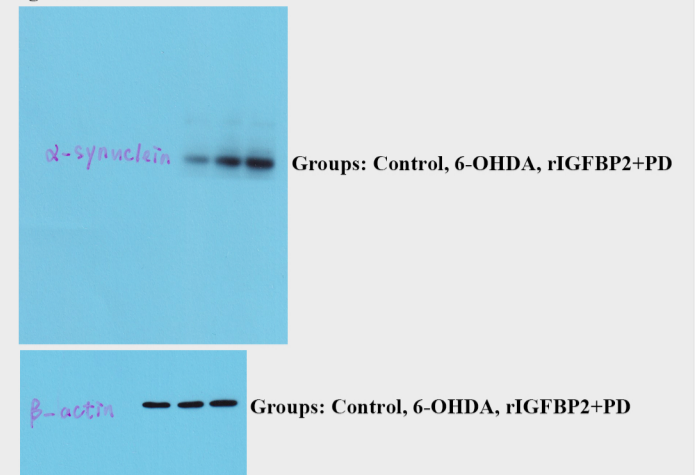

Figure 3D

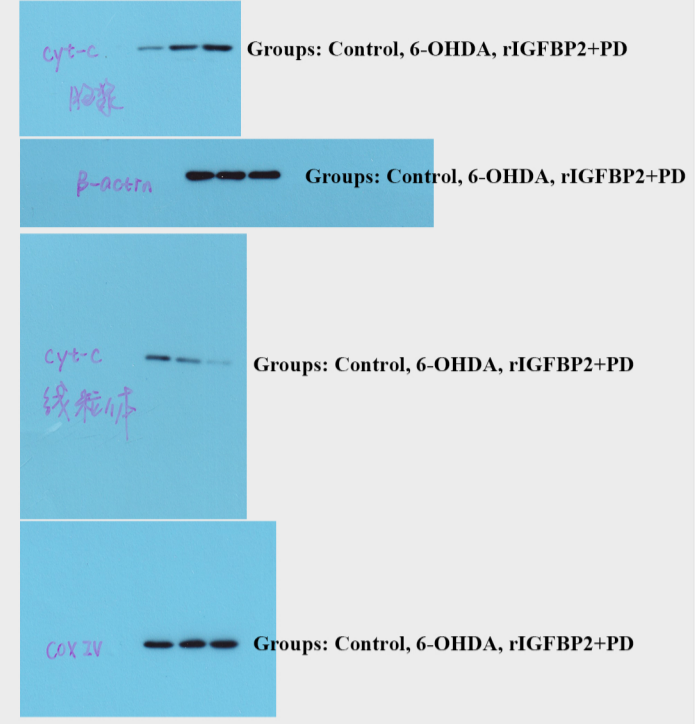

Figure 4A

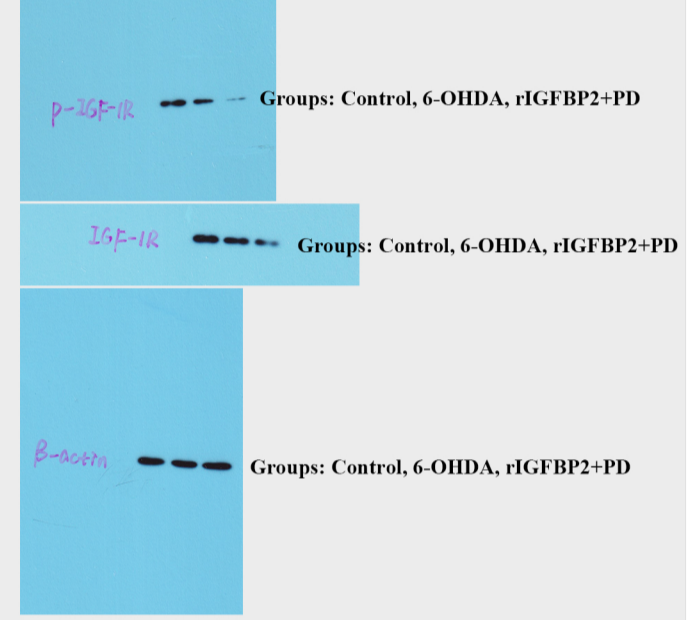

Figure 4B

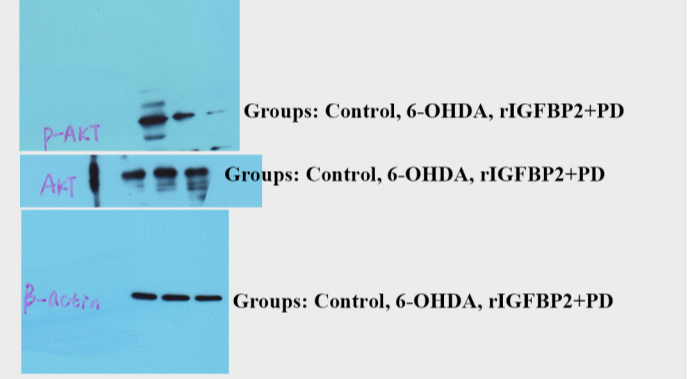

Figure 5D

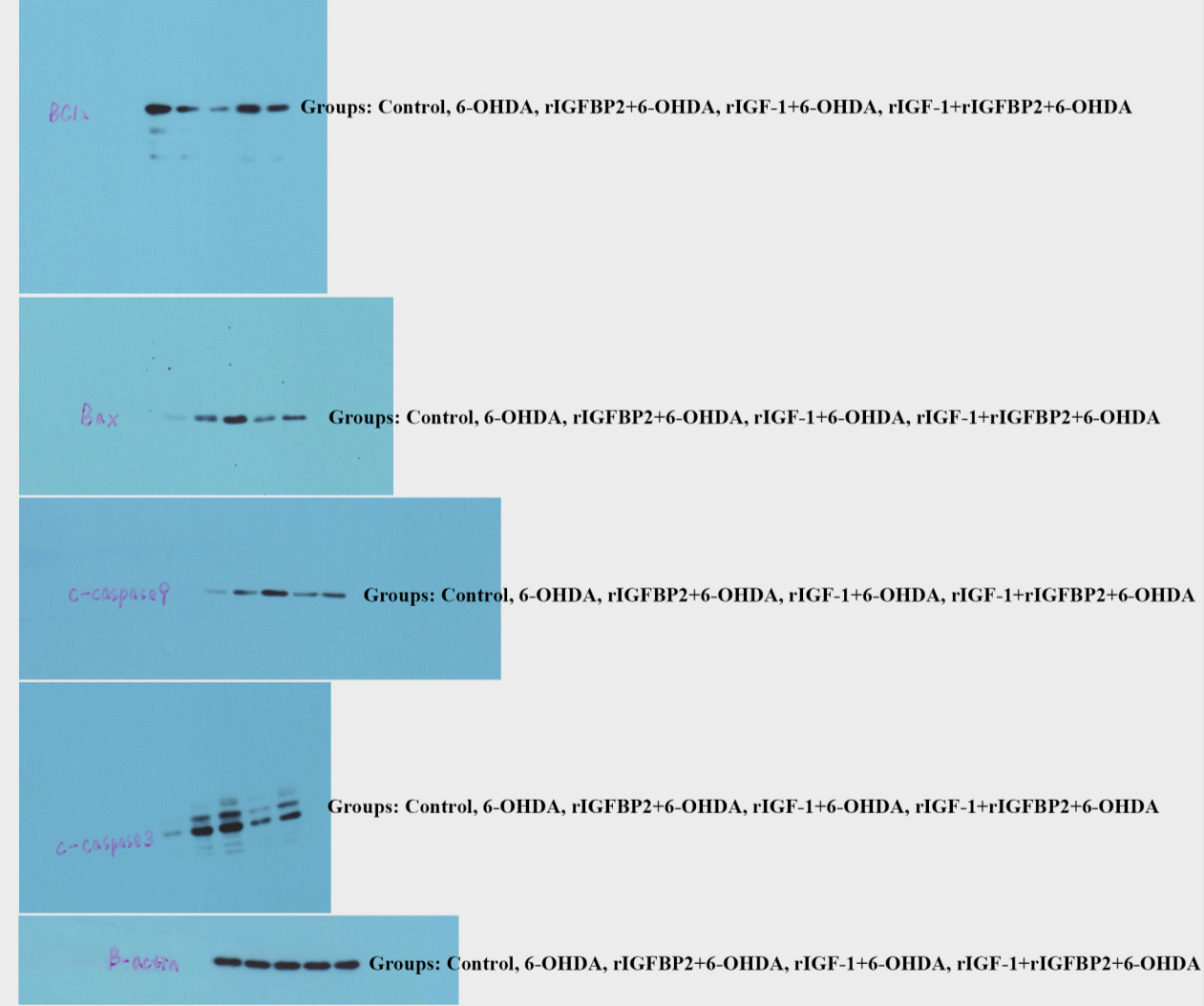

Figure 6C

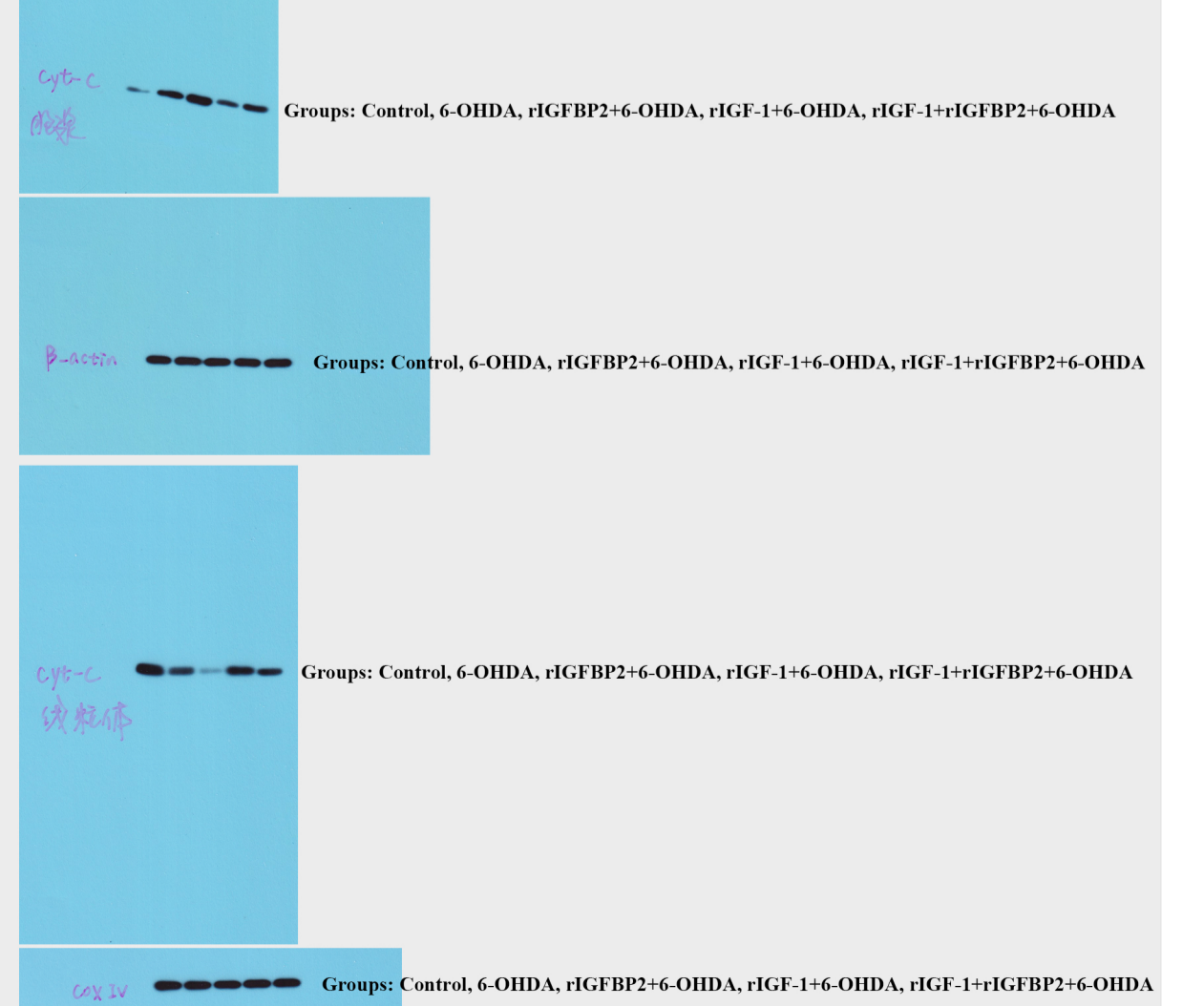

Supplement: Supplementary file 2 — Data S1. [file CNS-30-e70076-s001.pdf]
